# Chlamydophila pneumoniae induces expression of Toll-like Receptor 4 and release of TNF-α and MIP-2 via an NF-κB pathway in rat type II pneumocytes

**DOI:** 10.1186/1465-9921-6-51

**Published:** 2005-06-03

**Authors:** Heide Wissel, Christian Schulz, Petra Koehne, Ekkehard Richter, Matthias Maass, Mario Rüdiger

**Affiliations:** 1 Clinic for Neonatology, Campus Charité Mitte, Schumannstr. 20–21, D-10098 Berlin, Germany; 2 Clinic for Neonatology, Campus Charité Virchow-Klinikum, Berlin, Germany; 3 Department of Cell Biology, Institute of Biology, Humboldt-University Berlin, Germany; 4 SALK Microbiology, Salzburger Landeskliniken, Muellner-Hauptstr. 56, A-5020 Salzburg, Austria; 5 Clinic for Neonatology, Medical University Innsbruck, Austria

**Keywords:** Chlamydophila (Chlamydia) pneumoniae, rat type II pneumocytes, TLR4, NF-κB, cytokines

## Abstract

**Background:**

The role of alveolar type II cells in the regulation of innate and adaptive immunity is unclear. Toll-like receptors (TLRs) have been implicated in host defense. The purpose of the present study was to investigate whether Chlamydophila pneumoniae (I) alters the expression of TLR2 and/orTLR4 in type II cells in a (II) Rho-GTPase- and (III) NF-κB-dependent pathway, subsequently (IV) leading to the production of (IV) pro-inflammatory TNF-α and MIP-2.

**Methods:**

Isolated rat type II pneumocytes were incubated with C. pneumoniae after pre-treatment with calcium chelator BAPTA-AM, inhibitors of NF-κB (parthenolide, SN50) or with a specific inhibitor of the Rho-GTPase (mevastatin). TLR2 and TLR4 mRNA expressions were analyzed by PCR. Activation of TLR4, Rac1, RhoA protein and NF-κB was determined by Western blotting and confocal laser scan microscopy (CLSM) and TNF-α and MIP-2 release by ELISA.

**Results:**

Type II cells constitutively expressed TLR4 and TLR2 mRNA. A prominent induction of TLR4 but not TLR2 mRNA was detected after 2 hours of incubation with C. pneumoniae. The TLR4 protein expression reached a peak at 30 min, began to decrease within 1–2 hours and peaked again at 3 hours. Incubation of cells with heat-inactivated bacteria (56°C for 30 min) significantly reduced the TLR4 expression. Treated bacteria with polymyxin B (2 μg/ml) did not alter TLR4 expression. C. pneumoniae-induced NF-κB activity was blocked by TLR4 blocking antibodies. TLR4 mRNA and protein expression were inhibited in the presence of BAPTA-AM, SN50 or parthenolide. TNF-α and MIP-2 release was increased in type II cells in response to C. pneumoniae, whereas BAPTA-AM, SN50 or parthenolide decreased the C. pneumoniae-induced TNF-α and MIP-2 release. Mevastatin inhibited C. pneumoniae-mediated Rac1, RhoA and TLR4 expression.

**Conclusion:**

The TLR4 protein expression in rat type II cells is likely to be mediated by a heat-sensitive C. pneumoniae protein that induces a fast Ca^2+^-mediated NF-κB activity, necessary for maintenance of TLR4 expression and TNF-α and MIP-2 release through possibly Rac and Rho protein-dependent mechanism. These results indicate that type II pneumocytes play an important role in the innate pulmonary immune system and in inflammatory response mechanism of the alveolus.

## Background

The lung represents a site for the invasion of various bacteria or bacterial products. Along with alveolar macrophages, pulmonary epithelial cells are the first cells to be challenged by pathogenic microorganisms. The gram-negative bacterium Chlamydophila (Chlamydia) pneumoniae (C. pneumoniae) is an obligate intracellular pathogen causing acute and chronic pneumonia [1,2].

The Toll-like receptor (TLR)-family is an integral part of the innate immune system and recognizes conserved pathogen-associated molecular patterns (PAMPs) on microorganism. The interaction of TLRs with pathogen components initiates a signaling cascade that activates the adaptive immune response mechanisms which subsequently lead to inflammatory response and to the elimination of the pathogen [3].

TLRs are mainly expressed in professional immune cells in the alveolus. However, TLRs have also been found on type II pneumocytes [4-6] and could thus play an important role in the innate immune response at the alveolar surface area.

It is assumed that different TLRs recognize different classes of PAMPs [7]. TLR2 recognizes lipoproteins, peptidglycans and lipoteichoic acid. TLR4 is the receptor for lipopolysaccharide (LPS) and mediates the LPS signal transduction together with other molecules such as CD14, MD-2, myeloid differentiation factor 88 (MyD88), and so on [8].

Heat-shock protein (HSP) is one of the most phylogenetically conserved proteins in prokaryotes and eukaryotes [9]. Recent studies suggest, that chlamydial HSP stimulates innate immune and inflammatory responses by a TLR-mediated pathway, that is independent from LPS [10,11].

Recognition of PAMPs by TLR results in early host defense as well as in the activation of an inflammatory response pathway that involves mitogen-activated protein kinase (MAPK) and nuclear factor-kappaB (NF-κB). Furthermore, recognition of PAMPs induces the production of cytokines and stimulates the maturation of antigen-presenting cells [8,3].

Type II pneumocytes are responsible for the metabolism of alveolar surfactant and have recently been suggested to play an important role in the inflammatory response of the lung. Little is known about events that are induced by an interaction of bacteria with type II cells. We have recently shown that the contact of C. pneumoniae with microvilli of type II cells induces changes in cytoskeleton and leads to activation of NF-κB pathway [12]. Here, we tested whether C. pneumoniae stimulates expression of TLR2 and/orTLR4 in type II cells to induce the production of the pro-inflammatory cytokine TNF-α and chemokine MIP-2 via Rho-GTPase-and NF-κB-dependent pathways. The identification of the involved pathways is essential for the understanding of the innate immune defense mechanisms and pulmonary inflammatory response of the alveolus.

## Methods

### Cell culturing

C. pneumoniae strain TW183 was cultured and purified as described previously [13]. Briefly TW183 was grown to high titers in cycloheximide-treated HEp-2 cells. Infected monolayers were harvested from culture flasks and sonicated for 30 sec. Cellular debris was removed by centrifugation at 500 × g for 10 min at 4°C. Titer determination in cycloheximide-treated HEp-2 cells was performed with a thawed aliquot (in triplicate). Aliquots diluted with an equal volume of sucrose-phosphate-glutamate buffer supplemented with 10% foetal calf serum (FCS) were stored at -80°C until use. C. pneumoniae concentrations used were expressed as inclusion forming units (IFU)/ml. Type II pneumocytes were isolated from the lungs of adult male Wistar rats (body weight 120–140 g) according to Dobbs et al. [14].

Rat alveolar macrophages were separated from bronchial lavage fluid. The lungs were lavaged six times with phosphate-buffered saline (PBS) containing 0.2 mM EGTA (ethylene glycol tetraacetic acid). Cells were then collected by centrifugation at 230 × g for 10 min. The cell pellet was re-suspended in modified Eagle medium (MEM) (PAA laboratories GmbH, Linz, Austria).

Isolated type II cells were pre-incubated with the following substances: mevastatin (10 μM), also known as compactin, a specific inhibitor of the Rho-GTPase family; the calcium chelator BAPTA/AM (5 μM); SN50 (50 μg/ml), a cell-permeable peptide that inhibits the nuclear translocation of NF-κB/Rel complexes; SN50M (50 μg/ml), a cell-permeable control peptide; parthenolide (25 μM), an inhibitor of NF-κB through prevention of IκB phosphorylation (all chemicals were obtained from Calbiochem, Darmstadt, Germany) or MEM with corresponding concentrations of dimethyl sulfoxide (DMSO). Bacteria were treated with polymyxin obtained from Sigma, Deisenhofen, Germany. Thereafter, type II cells and macrophages in MEM in absence of FCS were incubated with or without C. pneumoniae with a multiplicity of infection (MOI) of 2 (2 × 10^6 ^IFU per 1 × 10^6 ^target cells) as used in previous experiments [12].

The viability of isolated alveolar macrophages or type II cells was assessed by trypan blue staining and was more than 95% or 98% respectively and remained above 94% after cell incubation with or without C. pneumoniae and different inhibitors.

Cell purity, estimated by hematoxylin staining, was more than 95% or 92% respectively.

### RNA extraction and Semi-Quantitative Polymerase Chain Reaction (PCR)

Cellular RNA was extracted from 5 × 10^5 ^type II cells using an RNeasy™ extraction kit (Quiagen, Hilden, Germany) according to the manufacturers instructions. RNA concentration was quantified by determining optical density at 260 and 280 nm (Ultraspec 2000 Spectrophotometer, Pharmacia Biotech, Freiburg, Germany) and adjusted to 100 pg/μl.

Semi-quantitative polymerase chain reaction was used to assess TLR2 and TLR4 mRNA expression under different conditions in rat alveolar type II cells. The mRNA expression levels of TLR2 and TLR4 genes were determined in total RNA from pooled samples of three independent experiments. Reverse transcription (RT) of 1 μg RNA into cDNA was performed by using random hexamers, AMV reverse transcriptase and 1× PCR buffer (20 mM Tris-HCL, pH 8.3, 50 mM KCl, 2 mM MgCl, 100 μg/ml bovine serum albumin). RT was performed at 42°C for 30 min followed by 95°C for 5 min to inactivate the reverse transcriptase enzyme. The cDNA was amplified by PCR in a total volume of 50 μl using 2.5 U Ampli-Taq DNA polymerase, 100 μM dATP, dCTP, dGTP, 50 μM dTTP and 0.5 μM of each primer in 1× PCR buffer. Incorporation of digoxigenin was performed by addition of 10 μM digoxigenin-11-dUTP to the PCR reaction mixture. For GAPDH housekeeping gene, PCR was performed with 25 cycles of 1 minute each at 95°C, 54°C and 72°C in a microprocessor-driven thermal cycler (Fa. Landgraf, Hannover, Germany). For TLR2 and TLR4 amplification, PCR was performed with 35 or 40 cycles of 1 minute each at 95°C, 65°C and 72°C.

Primer sequences for GAPDH were (GenBank BC059110; product size 303 bp):

5'-CAG TGC CAG CCT CTG CTC AT and

ATA CTC AGC ACC AGC ATC AT-3'.

Primer sequences for rat TLR2 were (GenBank AY151255; product size 199 bp):

5'-GTC CAT GTC CTG GTT GAC TGG and

GAT ACC ACA GCC CAT GGA AAT-3'.

Primer sequences for rat TLR4 were (GenBank NM_019178; product size 150 bp):

5'-GAG CCG GAA AGT TAT TGT GG and

AGC AAG GAC TTC TCC ACT TTC T-3'.

Products were stained with ethidium bromide and electrophoresed through a 1.5% agarose gel. The PCR products were transferred to nylon membranes by capillary blotting using 20× SSC as blotting buffer. The nylon membranes were fixed by UV light and the digoxigenin-UTP that had been incorporated into the PCR products was visualized by staining with anti-digoxigenin antibody conjugated to alkaline phosphatase [15]. Luminescence of the substrate (Lumigen™ PPD) was documented by short exposure to X-ray film.

Densitometric calculations of digital film images were performed with the analysis program Scion Image (Scion Corporation, Frederick, MD).

### Western blot analysis

Isolated type II cells were incubated with C. pneumoniae in suspension. Nuclear and cytoplasmatic extracts and membrane fractions were prepared from cells as reported previously [16,17]. The protein content of nuclear, cytoplasmatic and membrane fraction extracts was determined by Bradford assay using bovine serum albumin (BSA) as a standard method (Bio-Rad Laboratories protein assay kit, Richmond, USA). Protein fractions were stored at -80°C. TLR4 protein was assayed in membrane and cytoplasmatic extracts, IκBα protein in cytoplasmatic extracts, NF-κB p65 protein in nuclear extracts and Rac1 and RhoA protein in membrane extracts of type II cells by Western blotting. Proteins (40 μg or 60 μg for TLR4, 40 μg for IκBα, 10 μg for p65 and 40 μg for Rac1 and RhoA) from each sample were mixed with 2 × sodium dodecyl sulfate (SDS) sample buffer, heated at 95°C for 5 minutes, and separated by 10% (TLR4, p65), 12.5% (IκBα) and 15% (Rac1 and RhoA) SDS-polyacrylamide gels. The separated proteins were blotted onto nitrocellulose membranes. Non-specific binding sites were blocked with 10% nonfat dry milk in Tris-buffer saline (TBS)-0.05% Tween. Goat IgG anti-TLR4 (L-14) sc-16240, mouse monoclonal IgG anti-IκBα (H-4) sc-1643, rabbit IgG anti-NF-κB p65 (C-20) sc-372, goat IgG anti-Rac1 (T-17) sc-6084 and rabbit IgG anti-RhoA (119) sc-179 antibodies (from Santa Cruz Biotechnology, Heidelberg, Germany) were used for primary detection. Peroxidase-conjugated anti-goat IgG, anti-mouse IgG and anti-rabbit IgG antibodies (Dianova, Hamburg, Germany) were employed for secondary detection. The specific protein bands were visualized on film by enhanced chemiluminescence (ECL) (Amersham Biosciences, Inc, Piscataway, NJ) according to the manufacturers. The bands were quantified by scanning densitometry using a GS-710 Imaging Densitometer (Bio-Rad, Hercules, CA).

All other standard reagents and chemicals were of analytical grade and obtained from different suppliers.

### Immunocytochemistry and CLSM

The isolated type II cells were plated on glass cover slips for 3 hours at 37°C.

For TLR4-binding experiments, type II cells were incubated with and without C. pneumoniae for 10 min at 37°C, washed and then without permeabilization incubated with goat anti-TLR4 antibody for 60 min at 22°C.

Cells were pre-incubated with or without drugs or with corresponding concentrations of DMSO and incubated with C. pneumoniae as described by Wissel et al. [12,18]. Cells were fixed in 1% paraformaldehyde/250 mM Hepes (pH 7.4) and permeabilized with 0.04% saponin. For staining of TLR4 cells were incubated with goat anti-TLR4 antibody for 20 hours at 4°C. In experiments designed to block NF-κB activity, type II cells were pre-incubated with 20 μg/ml anti-TLR4 monoclonal antibody (HTA125, preservative free) or isotype control antibody (QBIOGENE-ALEXIS GmbH, Grünberg, Germany) for 30 min at 37°C before incubation with C. pneumoniae for 60 min at 37°C. To stain the NF-κB subunits within the cells, the following antibodies were used: mAb anti-IκBα and mAb anti-NF-κB p65 subunit, recognizing predominantly p65 only when IκBα is not bound to p65 (Chemicon, Hofheim, Germany) and incubated for 20 hours at 4°C. For staining of Rac1 and Rho protein cells were incubated with goat anti-Rac1 and rabbit anti-RhoA antibody for 2 hours at 22°C. After washing steps in PBS, cells were incubated with one of the following secondary antibodies: Alexa^® ^488 or Alexa^® ^594 anti-goat IgG, anti-mouse IgG labeled with Alexa^® ^488 or Alexa^® ^594, or with anti-rabbit IgG labeled with Alexa^® ^594 (Molecular Probes Europe BV, Leiden, Netherlands) for 2 hours at 22°C. To study the F-actin cytoskeleton, Alexa^® ^488-conjugated phalloidin (Molecular Probes) was used (2 hours at 22°C). Nuclear DNA was stained with 4',6-diamidino-2-phenylindole (DAPI) (Molecular Probes) for 20 minutes.

For TLR4-binding experiments samples were analyzed with a Leica CLSM (Leica Lasertechnik GmbH, Heidelberg, Germany), equipped with a krypton-argon laser as described previously [18]. Images were captured with a NPL Fluotar 40×/1.3 oil immersion objective. To visualize the cell boundaries, images were recorded simultaneously with bright-field contrast in relation with the channel system of CLSM in the equatorial plane of cells.

The triple staining was analyzed with a Zeiss laser scanning microscope LSM 510 META with Axiovert 200 M (Carl Zeiss Jena GmbH, Jena, Germany) as described previously [12]. Images were taken using a Plan-Apochromat 63×/1.4 oil immersion objective and fluorescence excitation at 488 nm (30 mW Ar-Laser), 543 nm (1 mW HeNe-Laser) and 405 nm (Diode Laser). To minimize signal crosstalk, a sequential scan with fast change of excitation lines was performed.

The semi-quantitative estimation of NF-κB subunits was made according to the methods described previously by Wissel et al. [12] and analyzed with Zeiss LSM 510 system with Axiovert microscope as described above. Thirty randomly chosen cells from six different regions were analyzed. Confocal settings were optimized and maintained for all images. Images were recorded with a scanning speed at 8s/frame with a resolution of 512 × 512 pixels. Serial sections of cells (z stack) at a depth of 0.5 μm were performed. Fluorescence intensity was determined on confocal images using a computer-based image analysis system equipped with MetaView™ software (obtained from Visitron Systems GmbH, Puchheim, Germany) by measuring the average intensity per pixel within a fixed-area circle placed over the relevant area of the cell.

### TNF-α ELISA and MIP-2 ELISA

Three-hour adherent type II cells and macrophages (2 × 10^6 ^cells/ml) on plastic dishes were incubated with or without C. pneumoniae for different periods of time. Adherent type II cells were pre-incubated with BAPTA/AM, SN50, SN50M or parthenolide followed by incubated with C. pneumoniae for 3 to 6 hours. Cell culture supernatants were collected and analyzed for TNF-α and MIP-2 secretion by enzyme-linked immunosorbent assay (ELISA). The levels of TNF-α production were determined using rat TNF-α Quanticine ELISA (R&D Systems Inc, Minneapolis, USA). MIP-2 concentration in the samples was determined using rat MIP-2 ELISA kit (Biosource Europe, Nivelles, Belgium) following the manufacturer' s instructions.

### Statistics

Statistical comparisons were performed by analysis of variance (ANOVA) with subsequent Fisher's protected least significant difference (PLSD) test. The level of significance was set at p < 0.05.

## Results

### C. pneumoniae increase the expression of TLR4 mRNA but not of TLR2 mRNA

**Figure 1:**
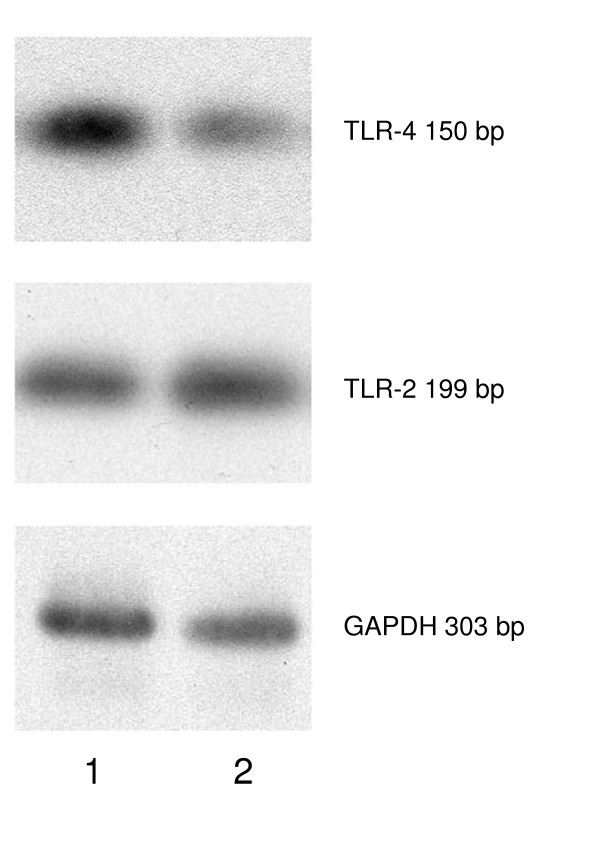
TLR4 mRNA expression in type II cells. Detection of TLR4 (upper lane) and TLR2 mRNA expression (middle lane) in type II cells after exposure to C. pneumoniae for 2 hours (1) and in non-infected control cells (2) by semiquantitative polymerase chain reaction. GAPDH (lower lane) served as internal control. After 2 hours of incubation with C. pneumoniae TLR4 mRNA expression in type II cells approximately doubled whereas TLR2 mRNA remained unchanged.

Incubation of rat type II pneumocytes with C. pneumoniae was associated with an increased expression of TLR4 mRNA (Figure 1). The mRNA concentration for TLR4 roughly doubled after 2 hours of incubation with C. pneumoniae compared to non-infected controls. The ratio between TLR4 mRNA signal and GAPDH signal was 0.8 in control type II cells and 1.4 after 2 hours of treatment with C. pneumoniae.

In contrast, 2 hours of incubation did not affect TLR2 mRNA expression, the ratio of TLR2 mRNA and GAPDH signal was 1.3 in control and 1.1 in C. pneumoniae treated cells (Figure 1).

The density of the shown GAPDH bands was homogenous for both infected and non-infected type II cells. The second GAPDH band showed a 0.89-fold expression compared to the first band.

### Effect of C. pneumoniae on TLR4 protein expression

**Figure 2:**
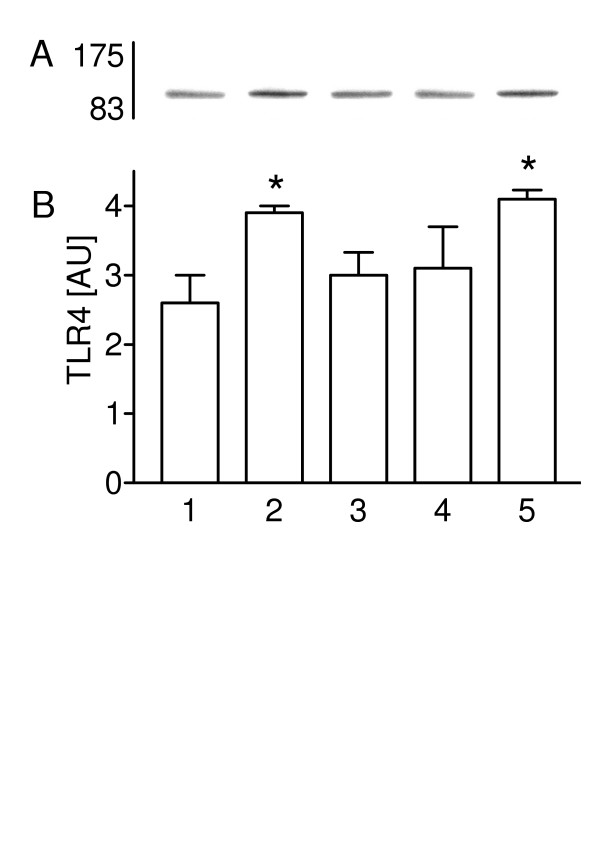
TLR4 protein expression in type II cells has two peeks. (A) The cytoplasm protein fraction of type II cells was subjected to SDS-PAGE and immunoblotting, non-infected cells (lane 1), 30 min (lane 2), 60 min (lane 3), 120 min (lane 4) and 180 min (lane 5) exposure with C. pneumoniae. (B) The TLR4 protein expression was estimated by densitometry. Values of n = 4 experiments are given as means ± SD in arbitrary units [AU]). Asterisk indicates a significant difference to controls (1), 60 min (3) and 120 min (4) (P <0.05).

To test whether C. pneumoniae contact rapidly increases the TLR4 protein expression and whether newly synthesized TLR4 protein is required, we first characterized C. pneumoniae-mediated TLR4 protein expression time-dependent in isolated rat type II cells. The presence of TLR4-protein was confirmed by Western blotting in non-infected and in type II cells after 30, 60, 120 and 180 minutes incubation with C. pneumoniae (Figure 2A and 2B). As quantified by scanning the chemiluminescence's bands from four different experiments. The TLR4 protein expression was highest at 30 and 180 min (1.6-fold increase versus control cells). After 60 min and 120 min incubation with C. pneumoniae the TLR4 expression did not differ from non-infected cells. These two peeks suggest that after 30 min contact with C. pneumoniae TLR4 protein is expressed constitutively at a post-transcriptional level whereas 3 hours incubation with bacteria mediated newly synthesized TLR4 protein expression.

TLR4 protein was only detectable with a goat polyclonal anti-TLR4 antibody (L-14), mapping an epitope within the extra cellular domain.

### Distribution of TLR4 in type II cells

Confocal imaging revealed rapid recycling of TLR4 from cytoplasm to the cell surface in response to C. pneumoniae incubation.

**Figure 3:**
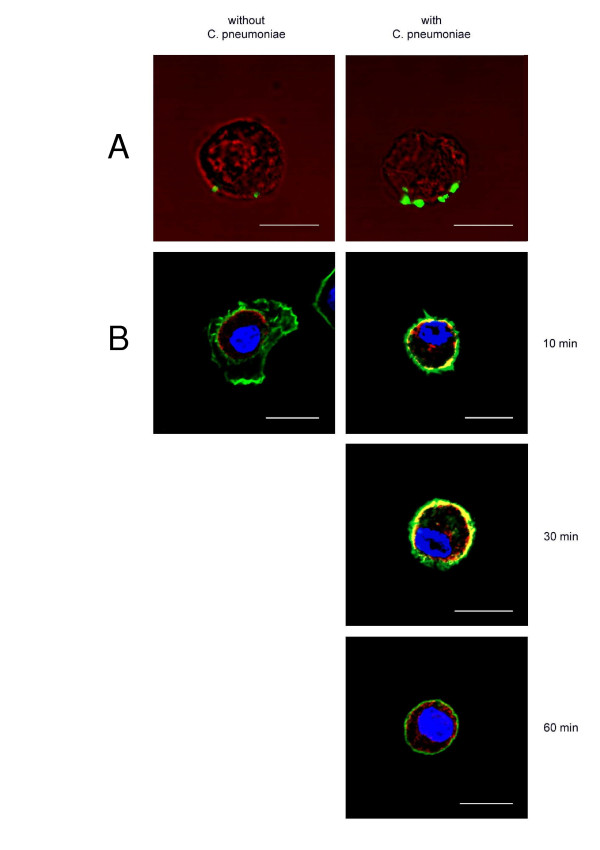
TLR4 is localized extra-and intracellularly in presence of C. pneumoniae. (A) Representative CLSM images after binding of C. pneumoniae at type II cells. Cells were without permeabilization stained with anti-TLR4 antibody that was detected by a secondary antibody coupled to Alexa488 (green). TLR4 is expressed at the external cell membrane. In un-infected cells no TLR4 expression was seen. Transmission images with pseudo-color red was used to highlight the outline of the cells. (B) Representative CLSM images of type II cells after incubation with C. pneumoniae at 37°C. Cells were after permeabilization stained with anti-TLR4 antibody that was detected by a secondary antibody coupled to Alexa594 (red) and labeled for F-actin with Alexa488-conjugated phalloidin (green). Nuclear DNA was stained with DAPI (blue). TLR4 mobilization to the external cell membrane reached a peak value after 10–30 min after exposure to C. pneumoniae. Areas of overlap between red and green are yellow, the bar equals 10 μm. Shown are representative examples, three more experiments gave similar results.

Binding-assays with C. pneumoniae incubation of type II cells demonstrated a TLR4 expression on the external membrane of the cells but not on non-infected cells (Figure 3A).

Time-dependent incubation with and without C. pneumoniae at 37°C, that was followed by fixation and permeabilization of the cells, revealed a TLR4 mobilization from the cytoplasm to the external cell surface that reached a peak value between 10–30 min after challenge with C. pneumoniae. The surface TLR4 expression started to decrease after 60 min in C. pneumoniae incubated cells. In non-infected cells TLR4 was partially co-localized with the intact F-actin cytoskeleton close to the cell membrane. A significant change in the F-actin cytoskeleton was found in cells after 30 and 60 minutes of C. pneumonia incubation (Figure 3B).

### Heat-inactivated C. pneumoniae have no effect on TLR4 expression

**Figure 4:**
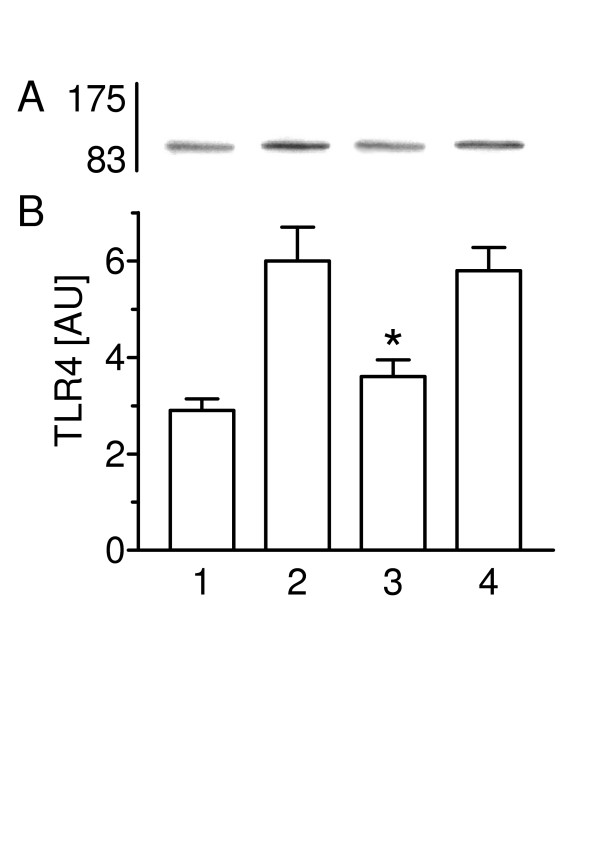
TLR4 expression is stimulated by a heat-sensitive component of C. pneumoniae. (A) TLR4 was visulalized by Western blot technique and (B) the expression determined by densitometry in non-infected cells (lane 1), non-treated C. pneumoniae (lane 2), heat-inactivated C. pneumoniae (lane 3) and polymyxin pre-treated bacteria (lane 4). Values of n = 4 experiments are given as means ± SD in arbitrary units. Asterisk indicates a significant difference to non-treated C. pneumoniae (2) and polymyxin pre-treated bacteria (4) (P < 0.0001).

Western blot analysis was used to determine the influence of protein components and/or LPS of C. pneumoniae on the expression of TLR4 in type II pneumocytes (Figure 4A and 4B). Incubation of type II cells with C. pneumoniae for 30 min stimulated TLR4 expression 2.1-fold when compared with non-infected controls. The TLR4 expression was significantly lower after incubation of cells with heat-inactivated bacteria (56°C for 30 min) than after 30 min of treatment with viable C. pneumoniae. Pre-treatment of C. pneumoniae with polymyxin B (2 μg/ml) did not alter the TLR4 expression.

### Anti-TLR4 antibodies inhibit C. pneumoniae-mediated NF-κB- activation

**Figure 5:**
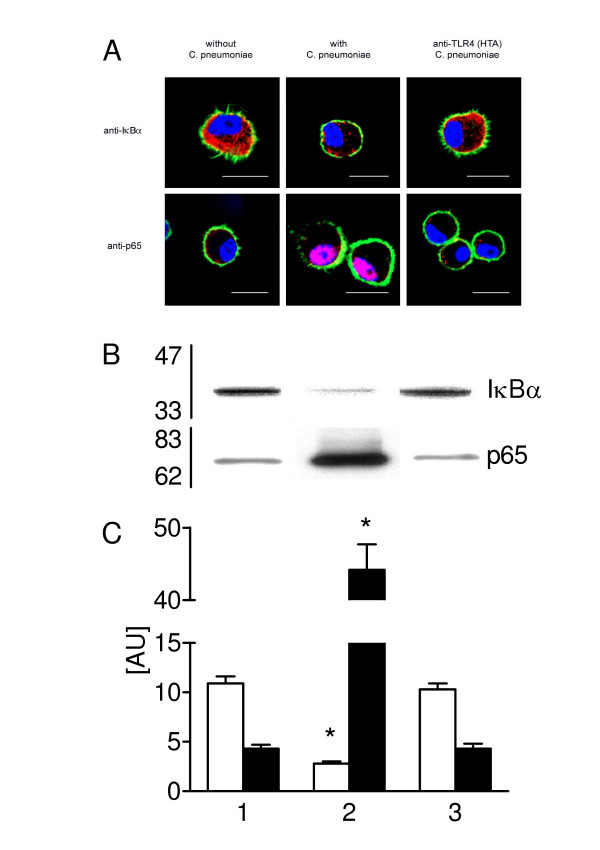
Inhibition of C. pneumoniae-mediated NF-κB activation with anti-TLR4 antibodies. (A) Representative CLSM images after incubation of type II cells pre-treated or not with anti-TLR4 antibodies followed by C. pneumoniae incubation for 30 min. Cells were stained with anti-IκBα antibody and anti-p65 antibody both detected by a secondary antibody coupled to Alexa594 (red), F-actin was stained with Alexa488-conjugated phalloidin (green) and nuclear DNA with DAPI (blue). In C. pneumoniae infected cells IκBα is decreased and p65 is localized in the nucleus. In cells that were treated with anti-TLR4 antibodies IκBα is not decreased and p65 is not localized in the nucleus. The effect on IκBα and NF-κB p65 was studied semi-quantitatively by CLSM as described in Materials and Methods. The C. pneumoniae-mediated IκBα degradation resulting in a 5.6-fold decrease versus control cells and NF-κB p65 translocation in the nucleus resulting in a 18-fold increase versus control cells. Areas of overlap between blue and red are pink, and between red and green are yellow, the bar equals 10 μm. (n = 5 experiments). (B and C) The amount of cytoplasmatic IκBα and of nuclear p65 of type II cells in controls (lane 1), incubated with C. pneumoniae for 30 min (lane 2) and pre-incubated with anti-TLR4 (HTA) antibodies prior to C. pneumoniae exposure for 30 min (lane 3) was quantified by Western blot analysis. Values of n = 3 experiments are given as means ± SD in arbitrary units. Open bars, IκBα; solid bars, p65. Asterisk indicates a significant difference to controls (1) and cells pre-incubated with anti-TLR4 antibodies (3) (P < 0.0001).

In experiments designed to block NF-κB activity, type II cells were pre-incubated with 20 μg/ml anti-TLR4 monoclonal antibodies (HTA125 or isotype control antibody) for 30 min and thereafter incubated with C. pneumoniae for 30 min. The effect on IκBα and NF-κB p65 was studied qualitatively (Figure 5A) and semi-quantitatively by CLSM. In non-infected type II cells IκBα was found in the cytoplasm and co-localized with the F-actin cytoskeleton. 30 min of bacterial contact caused a significant (p < 0.0001) decrease of the amount of IκBα in the cytoplasm when compared with control cells (15.9 ± 6 vs. 90.3 ± 16). Staining with anti-p65 antibody (recognizes p65 only when IκBα was not bound to p65) showed almost no activation of p65 in cytoplasm and nucleus in non-infected cells. Activated p65 was significantly (p < 0.0001) increased in the nucleus after C. pneumoniae contact (114 ± 10 vs. 6.3 ± 1.4 in control cells). Pre-treatment of cells with anti-TLR4 monoclonal antibodies, followed by C. pneumoniae incubation prevented IκBα decrease and p65 activation. Isotype control antibody had no effect on C. pneumoniae-mediated NF-κB activation (results not shown).

The amount of cytoplasmatic IκBα and of nuclear p65 was measured by Western blot analysis (Figure 5B and 5C). In comparison to control cells a 4-fold decrease in the amount of IκBα protein in the cytoplasm and a 10-fold increase of p65 in the nucleus was found 30 min after C. pneumoniae contact. In cells pre-treated with 20 μg/ml anti-TLR4 (HTA) antibodies, followed by C. pneumoniae incubation for 30 min neither C. pneumoniae-mediated IκBα degradation in the cytoplasm nor NF-κB activation of p65 in the nucleus was found.

### BAPTA-AM, parthenolide and SN50 suppress TLR4 mRNA and protein

**Figure 6:**
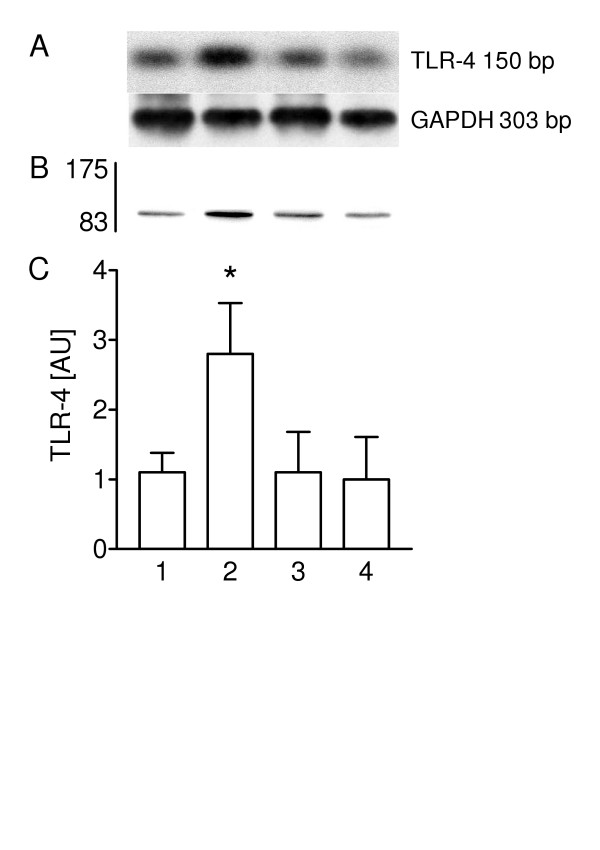
BAPTA-AM, parthenolide and SN50 inhibited C. pneumoniae-mediated TLR4 mRNA and protein expression. (A) Detection of TLR4 (upper lane) and GAPDH mRNA expression (lower lane) in type II cells by semi-quantitative polymerase chain reaction. The C. pneumoniae-induced TLR4 mRNA increase (2) returned to control values (1) after combined exposure to BAPTA-AM (3). Parthenolide (4) exerted an even stronger inhibitory activity and reduced TLR4 mRNA expression below baseline values. (B) Type II cells in suspension controls (lane 1) incubated with C. pneumoniae for 3 hours (lane 2), pre-incubated with BAPTA-AM (lane 3) and SN50 (lane 4) prior to C. pneumoniae exposure were prepared for Western blot analysis of TLR4 protein. (C) Their protein expression was determined by densitometry. Values of n = 3 experiments are given as means ± SD in arbitrary units. Asterisk indicates a significant difference to control (1), BAPTA-AM (3) and SN50 (4) (P < 0.05).

As shown in Figure 6A, mRNA expression for TLR4 approximately doubled in type II cells exposed to C. pneumoniae for 3 hours compared to controls. The TLR4 mRNA concentration remained at baseline values in BAPTA-AM and SN50 pre-incubated type II cells followed by 3 hours incubation with C. pneumoniae. The ratio between TLR4 mRNA signal and GAPDH signal was 1.7 in control cells, 1.0 in C. pneumoniae treated cells, 1.6 in BAPTA-AM pre-treated and infected cells and 1.8 in parthenolide pre-treated and infected cells. The density of the shown GAPDH bands was homogenous with GAPDH bands showing a 0.91, 0.98 and 0.84 fold expression compared to the first band, respectively.

The amount of TLR4 protein was analyzed by Western blot (Figure 6B) and quantified by scanning the chemiluminescence's bands from three different experiments (Figure 6C). TLR4 protein expression exposed to C. pneumoniae for 3 hours was 2.5-fold higher than in control cells. In contrast, the TLR4 protein expression in BAPTA-AM and SN50 pre-incubated type II cells followed by 3 hours of incubation with C. pneumoniae remained at control levels.

### C. pneumoniae-induced secretion of TNF-α and MIP-2 is NF-κB dependent

Tables 1 and 2 show that C. pneumoniae stimulate the release of TNF-α and MIP-2 from type II cells in a time-dependent manner. TNF-α concentration in the supernatant increased after 3–6 hours incubation with bacteria, and remained constant over the next 12 hours. The MIP-2 concentration in the supernatant continuously increased after bacterial incubation. To rule out a stimulatory effect due to contamination by macrophages (isolated type II cells contain less than 10% macrophages), the release of TNF-α and MIP-2 from isolated macrophages obtained from rat bronchial lavage fluid was measured. As tables 1 and 2 show, an estimated contamination of 10% macrophages would not influence the TNF-α or MIP-2 concentration in the supernatant. Pre-incubation of cells with BAPTA-AM, parthenolide and SN50 suppressed TNF-α and MIP-2 production (Table 3).

**Table 1:** TNF-α release in C. pneumoniae infected type II cells

	Type II cells + C. pneumoniae	Macrophages + C. pneumoniae	Type II cells – 10% macrophages
0 min	7 ± 1	13.7 ± 2.7	5.6
30 min	21 ± 7.5	69.5 ± 11.3	14
1 hour	109 ± 22	174 ± 9	92
2 hours	183 ± 29	303 ± 69	153
3 hours	656* ± 103	661§ ± 41	590
6 hours	1006** ± 74	1070§S ± 58	899
12 hours	996 ± 294	1038 ± 207	893

Mean values are given in pg/ml ± SD of three independent experiments.

* P < 0.0001 vs. 0–2 and 6–12 hours + C. pneumoniae

** P < 0.0001 vs. 0–3 hours + C. pneumoniae

§ P < 0.0001 vs. 0–2 and 6–12 hours + C. pneumoniae

§§ P < 0.0001 vs. 0–3 hours + C. pneumoniae

**Table 2:** MIP-2 release in C. pneumoniae infected type II cells

	Type II cells + C. pneumoniae	Macrophages + C. pneumoniae	Type II cells – 10% macrophages
0 min	160 ± 21	231 ± 22	137
30 min	210 ± 93	439 ± 49	166
1 hour	509 ± 76	1390 ± 329	370
2 hours	2405 ± 486	2715 ± 587	2134
3 hours	3367 ± 262	3600 ± 106	3007
6 hours	5274 ± 1095	11255 ± 2498	4149
12 hours	16179* ± 4577	24511§ ± 5161	13728

Mean values are given in pg/ml ± SD of three independent experiments.

* P < 0.0001 vs. 0–6 hours + C. pneumoniae

§ P < 0.0001 vs. 0–6 hours + C. pneumoniae

**Table 3:** BAPTA-AM, SN50 and parthenolide inhibited TNF-α and MIP-2 release in C. pneumoniae infected type II cells

			BAPTA-AM	SN50	SN50M	parthenolide
C. pneumoniae	-	+	+	+	+	+
TNF-α (3 h)	22.2* ± 7.5	703.4 ± 91	155.5* ± 30.7	122.6* ± 41	705.8 ± 65	125* ± 15.2
MIP-2 (6 h)	211* ± 28	4516 ± 182	2725* ± 413	2436* ± 253	3648 ± 49	1602* ± 24

Mean values are given in pg/ml ± SD of four independent experiments. Asterisk indicates a significant difference (P < 0.0001) compared to C. pneumoniae infected type II cells (column 2) or C. pneumoniae infected type II in the presence of SN50M (column 5).

### Rho-GTPases are involved in C. pneumoniae-mediated TLR4 protein expression

**Figure 7:**
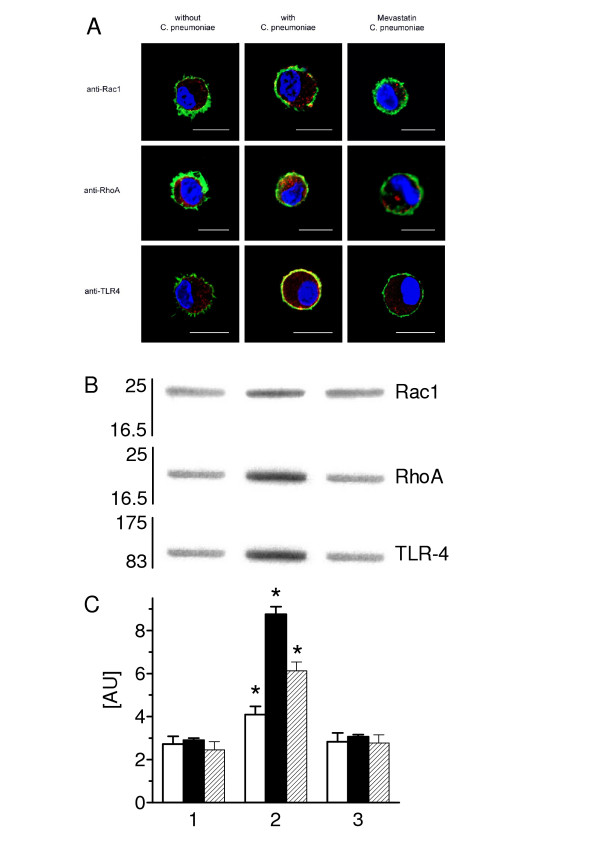
Mevastatin inhibited C. pneumoniae-mediated Rac1, RhoA and TLR4, expression. (A) Representative CLSM images of type II cells pre-treated and non-treated with mevastatin followed by C. pneumoniae incubation for 30 min. Detection with antibody for Rac1, RhoA and TLR4 followed by Alexa594-labeled secondary antibody (red). Labelled for F-actin with Alexa488-conjugated phalloidin (green) and nuclear DNA with DAPI (blue). The red labeled Rac1, RhoA and TLR4 appear yellow when co-localized with green labeled F-actin. The bar equals 10 μm. (B) Rac1, RhoA and TLR4 were visualized by Western blot technique and (C) their expression in membrane fractions was determined by densitometry in control cells (lane 1), C. pneumoniae incubated cells for 30 min in absence (lane 2) and in presence of mevastatin (lane 3). The values are means ± SD in arbitrary units of n = 3 experiments. Open bars, Rac1; solid bars, RhoA; hatched bars, TLR4. Asterisk indicates a significant difference compared to controls (1) and mevastatin (3) (P < 0.05).

To investigate whether C. pneumoniae-mediated TLR4 protein expression interferes with the F-actin-mediated Rho-GTPase activation, we examined the Rac1 and Rho protein expression after C. pneumoniae incubation for 30 min by CLSM. C. pneumoniae resulted in an increased expression of membrane-bound Rac1 and RhoA (Figure 7A). Furthermore, we tested the ability of mevastatin to down-regulate Rac1 and RhoA protein expression. As shown in Figure 7A mevastatin treatment reduced C. pneumoniae-mediated expression of both proteins. In non-infected type II cells, pre-treatment with mevastatin caused a destruction of the F-actin cytoskeleton (results not shown). We also observed that C. pneumoniae-induced stimulation of TLR4 can be decreased by mevastatin pre-treatment (Figure 7A).

Next, the Rac1, RhoA and TLR4 expression was examined by Western blotting of the membrane fractions of cells incubated with C. pneumoniae for 30 min in absence and presence of mevastatin (Figure 7B and 7C). As shown, C. pneumoniae resulted in a greater expression of Rac1 (1.5-fold), RhoA (3-fold) and TLR4 (2.5-fold) versus control cells. Mevastatin reduced C. pneumoniae-mediated Rac1, RhoA and TLR4 expression to control values.

## Discussion

Toll like receptors are crucial in mediating cellular responses to microbial components. The extra cellular portion of the TLR is directly involved in the recognition of various pathogens. C. pneumoniae are known to enter and persist in humane type II pneumocytes in chronic but not acute pneumonia [2]. The role of TLR in the pulmonary host defense system is unknown.

The present study on the interaction of type II pneumocytes with C. pneumoniae helps to understand the role of TLR4 within the innate immunity and inflammatory response mechanism of the lung. The study demonstrates that TLR2 and TLR4 mRNA is expressed constitutively in isolated rat type II cells. Incubation of alveolar type II cells with C. pneumoniae for 2 hours increased TLR4 but not TLR2 mRNA expression. Thus, TLR4 expression of rat type II cells is highly responsive to C. pneumoniae.

TLR4 is localized in the cell membrane and cytoplasm of type II cells. Incubation of type II cells with C. pneumoniae increased the expression of TLR4 at the external cell membrane surface within 10–30 min. Whereas the expression of TLR4 reached a peak value 30 min after C. pneumoniae contact, the TLR4 expression was less prominent after 60–120 min of incubation. An incubation time of 180 min was again associated with a high TLR4 expression. The data suggests, that constitutively expressed TLR4 in type II pneumocytes is rapidly responding to C. pneumoniae contact and mediates cellular responses to C. pneumoniae components at a post-transcriptional level. Thus, it seems as if C. pneumoniae is captured by constitutively expressed receptors very rapidly. To enable further binding of C. pneumoniae components, newly synthesized TLR4 is required. The decreased TLR4 expression due to C. pneumoniae binding at the cellular surface and the subsequent up-regulation of TLR4 expression could represent a barrier to prevent uncontrolled stimulation.

Various studies show that TLR4 is constitutively expressed in distinct human alveolar and bronchial epithelial cells. Lipopolysaccharide (LPS) stimulates the activation of TLR4 signaling in human alveolar type II-like (A549) and tracheo-bronchial (BEAS-2B, 16-HBE, and NT-1) epithelial cell lines [6]. Furthermore, LPS increased the expression of TLR2 and TLR4 mRNA and protein in human primary type II pneumocytes [5]. In normal human lung TLR2 mRNA and protein is mainly expressed in type II cells [4]. Whereas TLR4 is generally believed to be the receptor that mediates LPS responses [19], TLR2 mediates the inflammatory response to components of gram-positive bacteria and mycobacteria. However, TLR2 also mediates LPS-induced cellular signaling [20]. Different cells are activated by C. pneumoniae or cellular chlamydial components in a TLR2 and/or TLR4-dependent pathway [10,11,21-23].

Our data demonstrate that the TLR4 protein expression in rat type II cells is independent of chlamydial LPS. In addition, our results with heat-inactivated bacteria implicate that TLR4 expression is most likely mediated by a heat-sensitive chlamydial protein. C. pneumoniae and LPS free components of chlamydial heat shock protein 60 (cHSP60) were described to trigger inflammatory and cytokine responses in a TLR2-or TLR4-dependent way [10,21,23,24]. It can be speculated that the TLR ligand is a protein component of the outer membrane of C. pneumoniae, with cHSP60 being a possible candidate.

One regulator of TLR signaling is the NF-κB transcription factor, which controls the expression of many genes involved in the inflammatory response [3]. In vivo studies showed, that LPS induces NF-κB activation of type II cells [25,26]. The interaction of TLR2 and TLR4 with HSP60 triggers a signal cascade of protein kinase p38 and JNK1/2, mitogen-activated protein kinases ERK1/2 and IκB kinase (IKK) [11,27,28], that subsequently leads to NF-κB activation. We have recently shown that C. pneumoniae contact with the type II cell membrane activates the NF-κB pathway [12]. In the present study blockade of the TLR4 receptor at the cell membrane level reduced the C. pneumoniae-induced NF-κB activation in type II cells. Thus, results are in accordance with studies on mouse macrophages and human endothelial cells showing, that activation of NF-κB by chlamydial heat shock protein 60 (cHSP60) requires TLR4 [10].

We demonstrate that C. pneumoniae-mediated TLR4 mRNA and protein expression is attenuated by the calcium chelator BAPTA-AM and by inhibition of nuclear factor NF-κB with parthenolide or SN50. LPS-mediated TLR2 mRNA up-regulation in murine alveolar macrophages was attenuated by inhibition of NF-κB with sulfasalazine or SN50 [29].

BAPTA-AM, SN50 and parthenolide inhibited C. pneumoniae-mediated TNF-α and MIP-2 release in rat type II pneumocytes. It can be concluded that the calcium-mediated NF-κB activation is required for the transcriptional induction of TNF-α and MIP-2. Our findings support the idea that contact of C. pneumoniae with TLR4 triggers signal transduction and cytokine release. Contact of C. pneumoniae with TLR2 and TLR4 bone marrow-derived murine dendritic cells (BMDDC) leads to the translocation of NF-κB as well as secretion of TNF-α [22,24]. HSP60 from C. pneumoniae causes a TLR2-and TLR4-dependent increase in serum levels of MIP-2 in mice [11].

Intrapulmonary administration of Haemophilus influenzae in the mouse leads to TLR4-mediated TNF-α and MIP-2 expression by an activation of the NF-κB pathway in epithelial cells of the conducting airways. This suggests that the airway epithelia contribute to recognition of H. influenzae infection and induction of the innate immune response [30]. The role of alveolar type II cells in the regulation of lung inflammation is less clear. In vitro studies suggest that LPS-treatment of primary cultured type II cells from adult rat lungs leads to a TNF-α release, which initiates the synthesis and release of chemokines like MIP-2. Subsequently, the recruitment of inflammatory cells into the lung is promoted. These data indicate that a direct bacterial stimulation of alveolar epithelial cells can initiate the local host defense [31-33].

Recently, we demonstrated that NF-κB activation is involved in the calcium-mediated reorganization of the F-actin cytoskeleton after C. pneumoniae contact [12]. LPS induces the depolymerization of the cytoskeleton by mediating the TNF-α production in alveolar type II cells and may thus facilitate the migration of inflammatory cells [32]. Small GTPases of the Rho family (Rac and Rho) play an important role in several signal networks including actin cytoskeleton dynamics, transcriptional regulation, and membrane trafficking [34]. RhoA controls the assembly of actin and myosin stress fibers. Rac1 regulates the formation of membrane ruffles [35]. Data of the present study demonstrate that C. pneumoniae-induced TLR4 activation is Rac1 and RhoA protein mediated. Mevastatin, a specific inhibitor of the Rho-GTPase family, reduced the C. pneumoniae-mediated activation of TLR4, Rac1 and RhoA protein. In dendritic cells, TLR activation stimulates the antigen capture via F-actin remodeling [36]. Mevastatin (compactin) blocked the invasion of C. pneumoniae in Hep2 cells [37]. C. pneumoniae infection of vascular smooth muscle cells increased Rac1 and RhoA proteins and NF-κB expression that was suppressed by statin [38]. These results suggest a signaling cascade by which C. pneumoniae contact with the cell membrane leads to the activation of Rho family GTPases. The subsequent changes in actin cytoskeletal structure are essential for TLR4 expression and the activation of the NF-κB signaling pathway. The latter controls the expression of many genes involved in the innate immune system and adaptive inflammatory immune response.

We have recently shown, that C. pneumoniae changes the cytoskeleton of type II pneumocytes by NF-κB activation [12]. The current work demonstrates that NF-κB activation induces an inflammatory cascade that could support the local host defense. In healthy lungs, the pulmonary surfactant represents a barrier that can prevent the invasion of bacteria. However, C. pneumoniae inhibits the surfactant metabolism of isolated type II cells [18]. Thus, C. pneumoniae induce an immune response that prevents the invasion of C. pneumoniae into type II cells. Simultaneously, the impaired surfactant metabolism will enable bacteria to enter type II cells. The resulting net effect remains to be studied in vivo. Clinical data show, that no intracellular C. pneumoniae are found in patients with acute pneumonia, but in patients with chronic pneumonia [2]. It could be assumed that C. pneumoniae-induced alterations of type II cell cytoskeleton and surfactant metabolism will impair the host defense. Subsequently, more type II cells are invaded by C. pneumoniae, causing the clinical picture of chronic pneumonia.

## Conclusion

The TLR4 protein expression in rat type II cells is most likely stimulated by a heat-sensitive C. pneumoniae protein. The induction of a rapid Ca^2+^-mediated NF-κB activity is required for TLR4 expression and TNF-α and MIP-2release. A Rac1 and RhoA protein-dependent mechanism seems to be involved. These results implicate that TLR4 plays an important role in the innate pulmonary immune defense mechanism and in the inflammatory response of the alveolus.

## Authors' contributions

HW performed immunocytochemistry and CLSM, conceived and co-ordinated the study, acquired the data and wrote the manuscript. CS performed the ELISA analysis. PK performed the mRNA analysis. ER was involved in CLSM. MM participated in the C. pneumoniae strain TW183 culturing. MR was involved in the revision of the manuscript. All authors read and approved the final manuscript.
